# A novel signature for stratifying the molecular heterogeneity of the tissue-infiltrating T-cell receptor repertoire reflects gastric cancer prognosis

**DOI:** 10.1038/s41598-017-08289-z

**Published:** 2017-08-10

**Authors:** Manchao Kuang, Jieyao Cheng, Chengli Zhang, Lin Feng, Xue Xu, Yajing Zhang, Ming Zu, Jianfang Cui, Hang Yu, Kaitai Zhang, Aiming Yang, Shujun Cheng

**Affiliations:** 1 State Key Laboratory of Molecular Oncology, Department of Etiology and Carcinogenesis, National Cancer Center/Cancer Hospital, Chinese Academy of Medical Sciences and Peking Union Medical College, Beijing, China; 2 Division of Gastroenterology, Peking Union Medical College Hospital, Chinese Academy of Medical Science and Peking Union Medical College, Beijing, China

## Abstract

Many basic properties of the T-cell receptor (TCR) repertoire require clarification, and the changes occurring in the TCR repertoire during carcinogenesis, especially during precancerous stages, remain unclear. This study used deep sequencing analyses to examine 41 gastric tissue samples at different pathological stages, including low-grade intraepithelial neoplasia, high-grade intraepithelial neoplasia, early gastric cancer and matched adjacent tissues, to define the characteristics of the infiltrating TCRβ repertoire during gastric carcinogenesis. Moreover, to illustrate the relationship between the local molecular phenotype and TCR repertoire of the microenvironment, whole-genome gene expression microarray analysis of the corresponding gastric precancerous lesions and early gastric cancer tissues was conducted. Our results showed that the degree of variation in the TCR repertoire gradually increased during tumourigenesis. Integrative analysis of microarray data and the TCR repertoire variation index using the network-based Clique Percolation Method identified an 11-gene module related to the inflammatory response that can predict the overall survival of gastric cancer (GC) patients. In conclusion, our results revealed the multistage heterogeneity of tissue-infiltrating TCR repertoire during carcinogenesis. We report a novel way for identifying prognostic biomarkers for GC patients and improves our understanding of immune responses during gastric carcinogenesis.

## Introduction

Gastric cancer (GC) is the third leading cause of cancer death in both sexes worldwide^1^. Prior to the development of GC, especially intestinal GC, a prolonged precancerous stage characterized by the following well-defined sequential stages is observed: chronic active gastritis, chronic atrophic gastritis, intestinal metaplasia, and finally, dysplasia (also called intraepithelial neoplasia)^2–5^. According to the WHO classification of tumours of the digestive system^6^, low-grade intraepithelial neoplasia (LGIN) and high-grade intraepithelial neoplasia (HGIN) are considered gastric precancerous lesions (GPLs). A cohort study demonstrated that 2.1% of patients with LGIN progress to GC within 1 year, whereas this number increases to 24.9% for patients with HGIN^7^. In most countries, approximately 80% of patients are diagnosed during the advanced stages of the disease, which are associated with a 5-year survival rate of only 10%. Earlier detection dramatically improves the 5-year survival rate to 90%^8–11^. Despite extensive efforts in recent decades, effective treatments for advanced GC or specific diagnostic markers for GPLs and early gastric cancer (EGC) remain elusive.

Dominated by T-cell checkpoint inhibitor and chimeric antigen receptor (CAR) T-cell therapies, cancer immunotherapy has achieved remarkable clinical efficacy in patients with different types of cancer, including GC^12–15^. Specifically, a clinical trial of adoptive immunotherapy with tumour-associated lymphocytes combined with chemotherapy in advanced GC showed a survival benefit over chemotherapy alone^16^. GC development and progression are associated with multiple risk factors, and the function and distribution of tumour-infiltrating T lymphocytes (TILs) play crucial roles in this progression because T cells play an essential role in immune-mediated tumour surveillance. Moreover, the number or proportion of TILs is of great prognostic value in a variety of cancers^17^, and TILs directly targeting tumour-associated antigens could be important in the defence against tumours, which highlights potential clinical applications of immunotherapy. Therefore, the signature of TILs urgently needs to be systematically and comprehensively characterized to better understand of the mechanism of cancer immunotherapy and to discover predictive biomarkers that can be used to assess treatment response.

Recently, a deep sequencing-based TCR repertoire analysis revealed a panorama of various adaptive immune responses^18^. Among TCRs, largely consist of α and β chains^19^. Rearrangement of variable (V), diversity (D) and joining (J) segments in TCRβ generates the highly variable complementary determining region 3 (CDR3), which is critical for the specificity and affinity of antigen recognition. CDR3 shapes the spectrum of TCR diversity^20^ and allows T cells to target any endogenous or exogenous antigen^21^. Hence, TCR repertoire sequencing can be used to assess the immune responses of cancer patients.

Most studies have focused on differences in the TCR repertoire between tumour and healthy control tissues^22, 23^, whereas others have attempted to delineate the spatial heterogeneity of TILs^24–26^. However, the changes in the TCR repertoire during carcinogenesis, especially during the precancerous stages, have not yet been characterized. In our study, we used TCRβ sequencing to investigate tissue-infiltrating lymphocytes in GPLs, EGC and matched adjacent tissues. Moreover, gene expression was profiled in the corresponding GPLs and EGC samples using a whole-genome microarray. Overall, our study aimed to (1) assess dynamic changes in the TCR repertoire during gastric tumourigenesis and (2) determine the potential clinical value of variations in the TCR repertoire.

## Results

### High-throughput sequencing of TCR repertoires

To assess the immunogenicity of gastric precancerous lesion and EGC, we amplified and sequenced the TCRβ CDR3 regions of tissue-infiltrating T cells of 41 gastric tissues from 19 patients (Table 1). An average of 3,224,191 (range: 2,232,415–4,903,297) total TCRβ sequences were detected in each sample, and an average of 49,444 (range: 32,048–101,029) unique TCRβ sequences were detected in each sample (Supplementary Table S1).

**Table 1:** Study populations.

Sample ID	Sex	Age	Sample type			
			Adjacent mucosa	LGIN	HGIN	EGC
Patient 1	Male	45	√	√	√	
Patient 2	Male	58	√	√	√	
Patient 3	Male	60	√	√	√	
Patient 4	Female	61	√	√		
Patient 5	Male	54	√	√		
Patient 6	Female	60	√	√		
Patient 7	Female	74	√	√		
Patient 8	Male	55	√		√	
Patient 9	Female	71	√		√	
Patient 10	Male	46	√		√	
Patient 11	Male	56	√		√	
Patient 12	Male	55	√		√	
Patient 13	Male	54	√		√	
Patient 14	Female	61	√			√
Patient 15	Female	78	√			√
Patient 16	Male	57	√			√
Patient 17	Female	56	√			√
Patient 18	Male	71	√			√
Patient 19	Male	55	√			√

**Figure 1:**
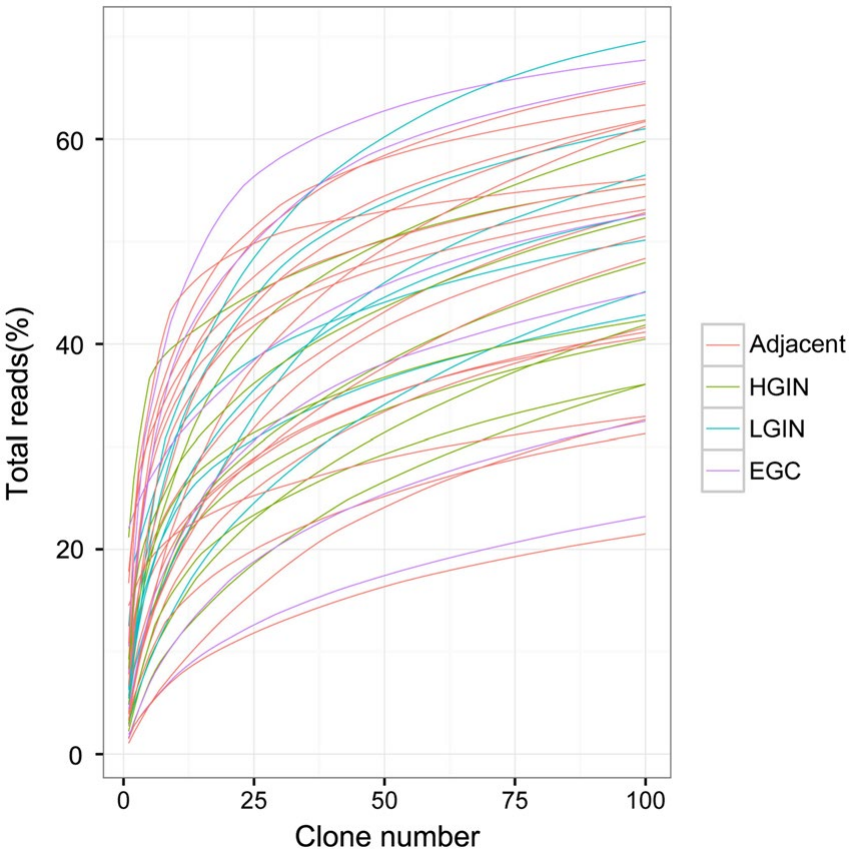
Characteristics of the TCR repertoire frequency distribution. The cumulative frequencies of the TOP100 in each sample are shown. The x-axis depicts the number of clones included (starting from the most expanded clones). The y-axis shows the cumulative percentage of TCRβ sequences that are covered by the included clones.

The percentages of T-cell clones with different frequencies are summarized in Table S2. The data suggested that the TCR clones showed a skewed frequency distribution, i.e., a tiny fraction of highly expanded clones. To further explore the configuration of the TCR repertoire, a cumulative frequency graph (Fig. 1) was generated for the “most expanded top 100 clones” (defined as TOP100) of each sample. The graph showed that the cumulative frequencies sharply increased; the average cumulative frequencies of the TOP100 for adjacent mucosa, LGIN, HGIN and EGC tissues were 48.74 ± 2.9%, 53.97 ± 3.5%, 45.82 ± 2.8% and 47.78 ± 7.2%, respectively. The cumulative frequencies of the TOP100 for all of the samples were almost 50%, which indicated that the entire TCR repertoire was dominated by a small fraction of clones. The cumulative frequencies of the TOP100 did not significantly differ between gastric lesions and adjacent tissues (Two-tailed paired t-test, p > 0.05; Supplementary Fig. S1). These findings indicated that the frequency distribution of the TCR repertoire during gastric tumourigenesis was not significantly changed.

Furthermore, Shannon-Wiener diversity index (ShannonDI) was applied to quantify the diversity of the T-cell clones in each sample. The results showed that T-cell clone diversity was not significantly different between gastric lesions and matched adjacent tissues (Two-tailed paired t-test, p > 0.05; Supplementary Fig. S2).

### TCR repertoires overlap between gastric lesions and matched adjacent tissues

**Figure 2:**
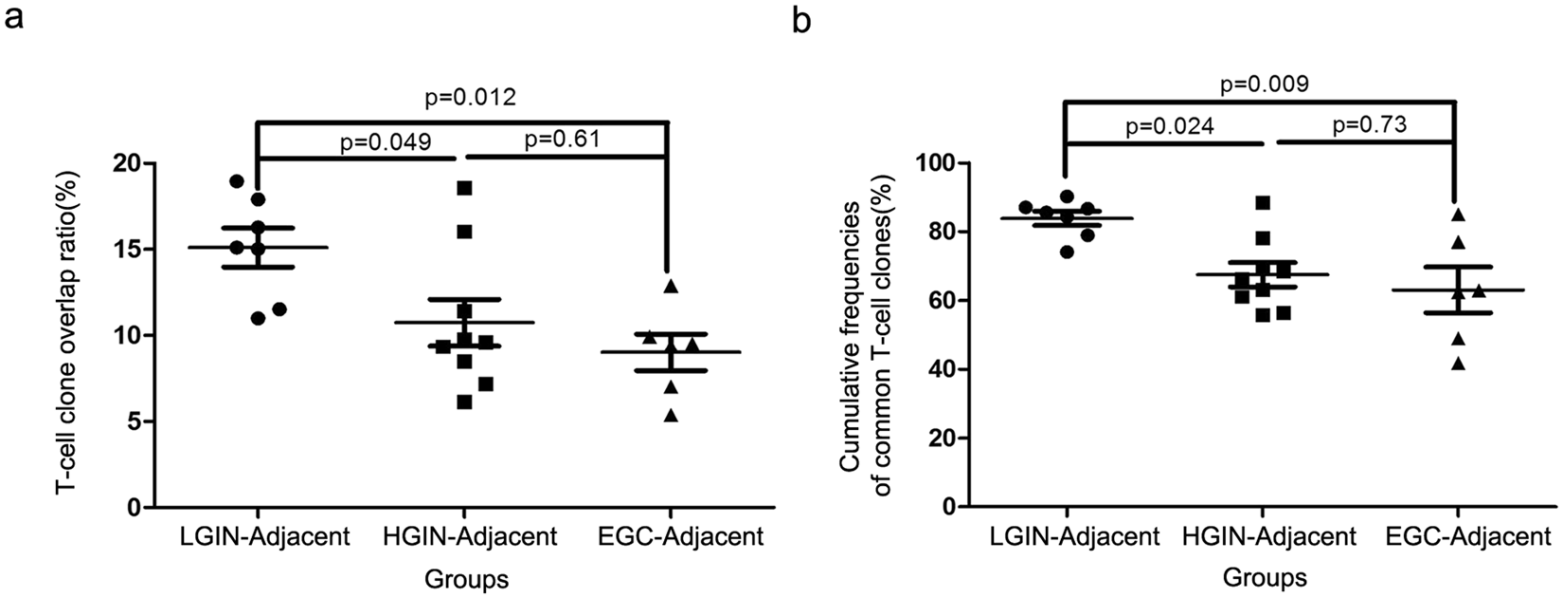
Comparison of the TCR repertoire overlap between gastric lesions and corresponding adjacent tissues. The T-cell clone overlap ratio (**a**) and the cumulative frequencies of common T-cell clones (**b**) between LGIN and adjacent tissues were significantly higher than HGIN-adjacent mucosa pairs and EGC-adjacent mucosa pairs. One-way ANOVA and Tukey’s post hoc multiple comparison test, p < 0.05.

We first identified the T-cell clones that were shared between gastric lesions and matched adjacent tissues to compare similarities and differences in TCR repertoires during gastric carcinogenesis. The average T-cell clone overlap ratio between LGIN and adjacent tissues was 15.11 ± 1.13%, higher than the ratio between HGIN and adjacent tissues (10.74 ± 1.35%) and that between EGC and adjacent tissues (9.02 ± 1.06%) (One-way analysis of variance (ANOVA), p = 0.011; Fig. 2A). The same result was obtained by analysing the cumulative frequencies of common T-cell clones. The average cumulative frequencies of common T-cell clones were 83.84 ± 2.08%, 67.48 ± 3.50% and 63.03 ± 6.64% for LGIN-adjacent mucosa pairs, HGIN-adjacent mucosa pairs and EGC-adjacent mucosa pairs, respectively (One-way ANOVA, p = 0.0069; Fig. 2B). These results demonstrated that the similarity of TCR repertoires between gastric lesions and adjacent tissues gradually decreased during gastric tumourigenesis.

### Overlap between the TOP100 of gastric lesions and the TCR repertoires of matched adjacent tissues

**Figure 3:**
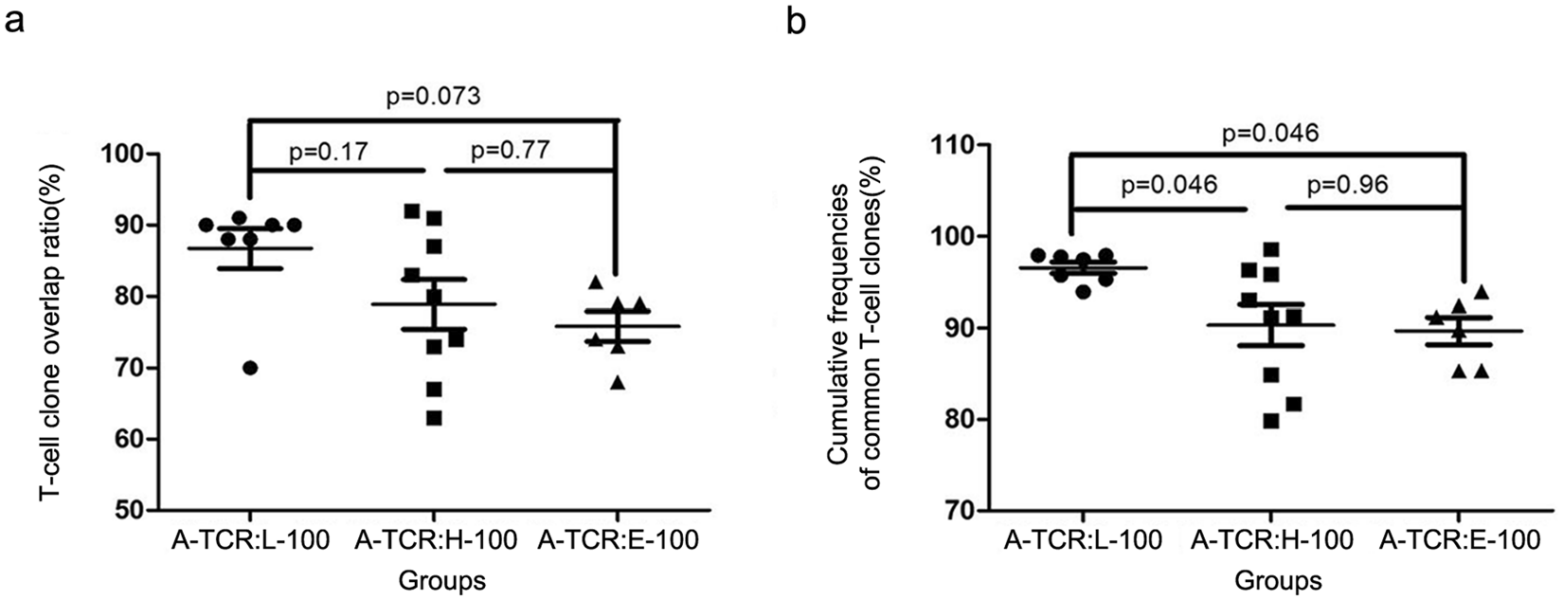
Comparison of the overlap between the TOP100 of gastric lesions and A-TCR. The T-cell clone overlap ratio (**a**) and the cumulative frequencies of common T-cell clones (**b**) between A-TCR and L-100 were significantly higher than those between A-TCR and H-100 and between A-TCR and E-100. One-way ANOVA and Tukey’s post hoc multiple comparison test, p < 0.05.

Due to the skewed frequency distribution of T-cell clones, the small fraction of highly expanded T-cell clones, which may dominate the TCR repertoires, require further exploration. Thus, we next analysed the overlap between the TOP100 of the LGIN, HGIN, and EGC subsets (named L-100, H-100 and E-100, respectively) and the TCR repertoires of the corresponding adjacent tissues (named A-TCR). The average T-cell clone overlap ratio between A-TCR and L-100 (86.71 ± 2.82%) was higher than the ratio between A-TCR and H-100 (78.89 ± 3.45%) and that between A-TCR and E-100 (75.83 ± 2.09%) (One-way ANOVA, p = 0.07; Fig. 3A); however, the p-value was marginally significant. The average cumulative frequencies of common T-cell clones gradually reduced during the stages of tumourigenesis. Specifically, the frequencies for the LGIN-adjacent mucosa pairs, HGIN-adjacent mucosa pairs and EGC-adjacent mucosa pairs were 96.57 ± 0.60%, 90.31 ± 2.23% and 89.67 ± 1.48%, respectively (One-way ANOVA, p = 0.027; Fig. 3B).

These results were consistent with the overall analysis of the T-cell clones. Moreover, the results showed that lesion antigen-associated T-cell clones were generated and accumulated with tumour progression and became highly expanded T-cell clones, which may play a key role in tumour immunity.

### The frequency similarity of “the top 100 common T-cell clones” between gastric lesions and adjacent tissues

**Figure 4:**
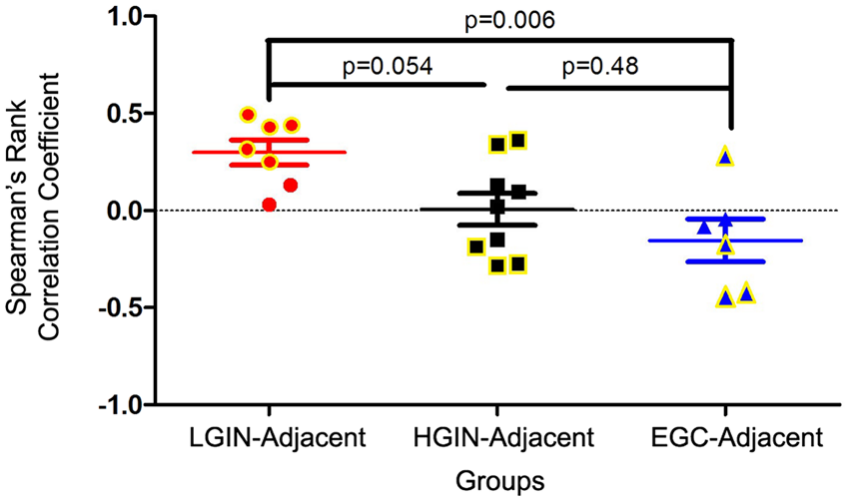
Correlation analysis of “the top 100 common T-cell clones” between gastric lesions and corresponding adjacent tissues. Specifically, LGIN and adjacent tissues showed a positive correlation, but the correlation between HGIN and adjacent tissues was heterogeneous; moreover, most EGC and adjacent tissues showed a negative correlation. One-way ANOVA and Tukey’s post hoc multiple comparison test, p < 0.05. Yellow indicates that the correlation was significant.

To further evaluate the changes in the frequency of common T-cell clones during gastric tumourigenesis, we used Spearman’s rank correlation test to analyse the relationship between “the top 100 common T-cell clones” (i.e., clones shared between gastric lesions and matched adjacent tissue; and at least in the TOP100 of either the gastric lesions or adjacent tissues) in gastric lesions and corresponding adjacent tissues. As shown in Fig. 4, LGIN and adjacent tissues exhibited a positive correlation, but the correlation between HGIN and adjacent tissues was heterogeneous. Moreover, most EGC and adjacent tissues showed a negative correlation (One-way ANOVA, p = 0.007). This result indicated that during tumourigenesis, not only did the ratios of common T-cell clones gradually decrease, but their frequencies also gradually changed, some high-frequency TCR clones turned into low-frequency ones, while some lesion specific clones abnormally amplified.

### Identification of genes correlated with the TCR repertoire variation index (TVI)

**Figure 5:**
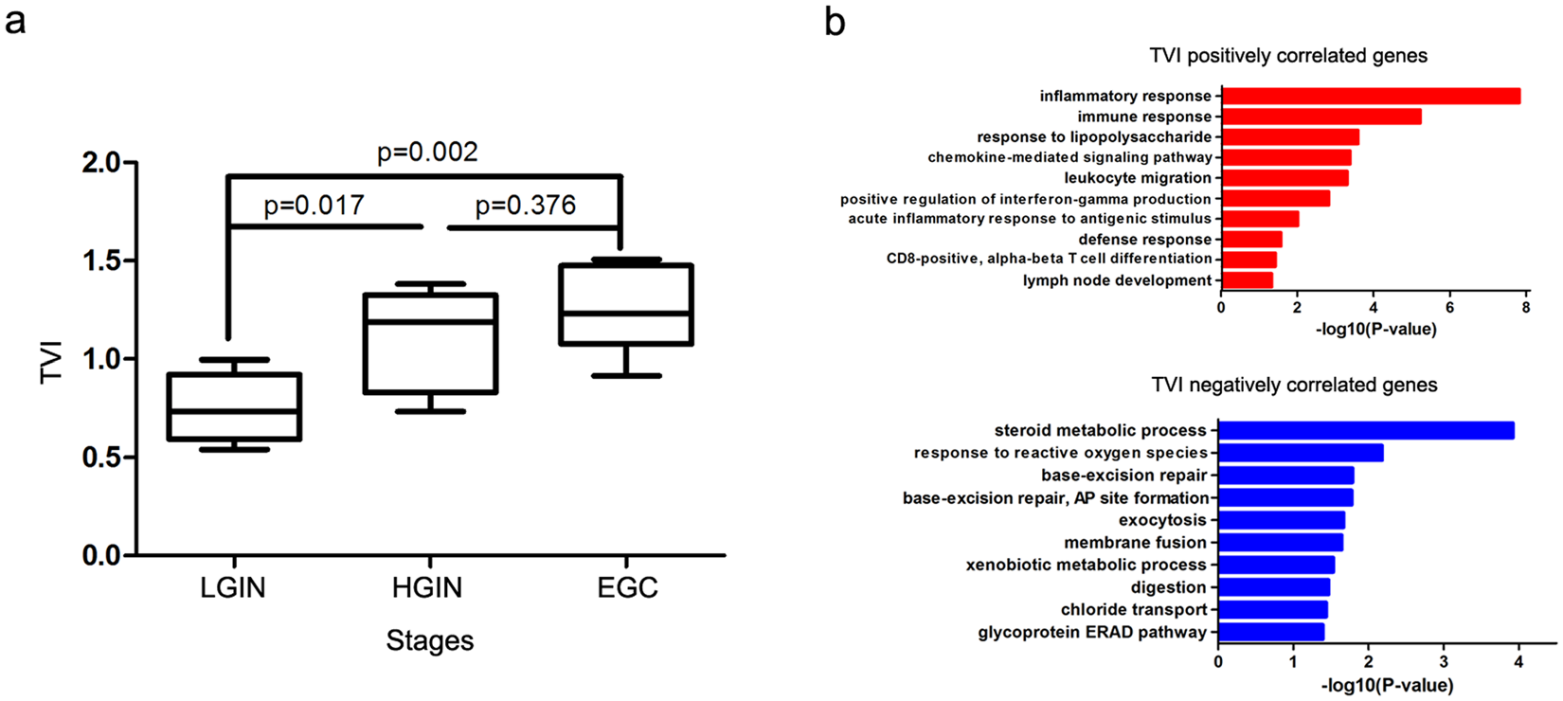
Identification of genes correlated with TVI. (**a**) The TVI gradually increased during gastric tumourigenesis. One-way ANOVA and Tukey’s post hoc multiple comparison test, p < 0.05. (**b**) Representative GO biological process terms for TVI-correlated genes.

To further evaluate the degree of variation in the TCR repertoire during gastric tumourigenesis, we calculated the TVI values. As shown in Fig. 5A, the TVI gradually increased during tumourigenesis (One-way ANOVA, p = 0.002). Then, the mRNA transcriptome profiles of 22 GPLs and EGC tissues were obtained using an Agilent microarray to represent the local molecular phenotype.

In order to identify the correlation between the TVI of the microenvironment and local molecular phenotype, Spearman’s rank correlation test was performed between the TVI values of TCRβ and the expression levels of all detectable genes in the microarray of GPLs and EGC tissues; 378 genes showed a significantly positive correlation, and 410 genes showed a significantly negative correlation (FDR < 0.05). Based on Gene Ontology (GO) enrichment analysis, these positively correlated genes are associated with inflammatory response, immune response, leukocyte migration and acute inflammatory response to antigenic stimulus (ontology: biological process), whereas negatively correlated genes are related to steroid metabolic process, response to reactive oxygen species, base-excision repair and exocytosis (ontology: biological process; Fig. 5B).

### A network-based method identified an 11-gene module that can predict the overall survival of GC patients

**Figure 6:**
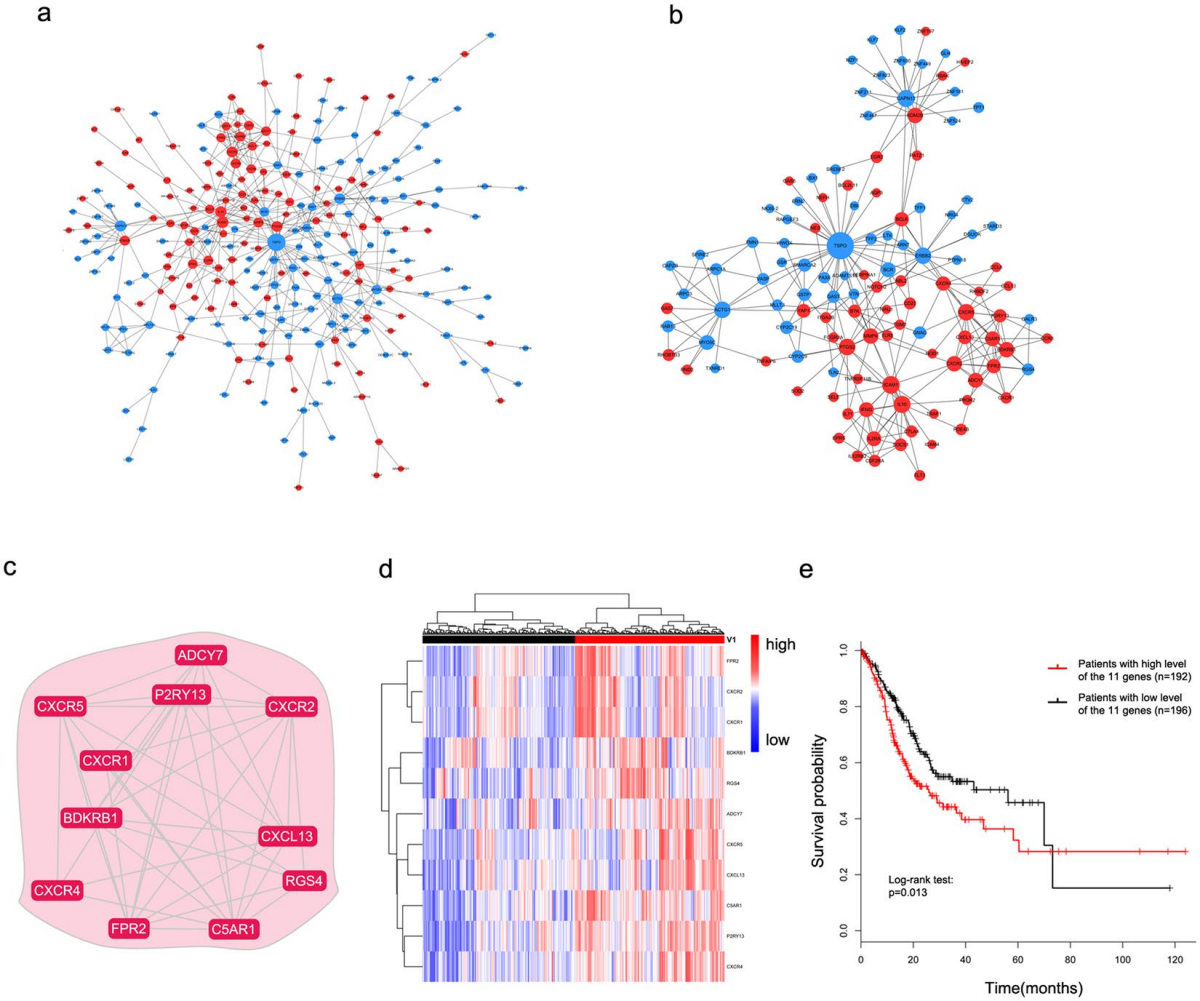
Identification of an 11-gene module related to the overall survival of GC patients using the network-based method. (**a**) The maximal subnetwork contained 265 nodes and 462 edges. (**b**) The hub network contained 12 nodes in the top 5%, with respect to degree and their first neighbour nodes. (**c**) The 11-gene module was identified using the Clique Percolation Method (k = 5). (**d**) The 388 TCGA gastric tumour patients were divided into two groups according to the expression levels of the 11 genes using unsupervised hierarchical clustering. The expression levels of these genes are illustrated as a colour spectrum, with red, white and blue representing high, medium and low expression, respectively. (**e**) Kaplan-Meier survival curves and log-rank tests were used to estimate the overall survival of the two patient groups, p < 0.05.

All 788 TVI-related genes were mapped to the STRING database to build the protein-protein interaction (PPI) pairs, and Spearman’s correlation coefficients were calculated to filter the PPI pairs. Finally, a maximal subnetwork with 265 nodes and 462 edges was generated (Fig. 6A). This network exhibited a scale-free connectivity (Power > 0.85), in accordance with the characteristics of the biological network (Supplementary Fig. S3). Subsequently, we selected the 12 hub nodes in the top 5% with respect to degree and their first neighbour nodes, to construct a hub network (Fig. 6B) and then used CFinder to detect cliques based on the Clique Percolation Method. Eventually, we found a module with 11 nodes (Fig. 6C), which had the highest stringency (k = 5). GO enrichment analysis showed that this 11-gene module was closely related to the inflammatory response and chemotaxis (Supplementary Table S3).

The prognostic significance of this TVI-related 11-gene module was tested in The Cancer Genome Atlas (TCGA) GC dataset. The 388 GC samples were divided into two groups by unsupervised hierarchical clustering according to the expression level of the 11 genes (Fig. 6D). We found that the 11-gene signature was closely related to the overall survival of GC patients (Log-rank test, p = 0.013; Fig. 6E). Those results indicated that these TVI-related genes might play crucial roles in determining the prognosis of cancer patients.

## Discussion

Cancer is a genetic disease caused by the accumulation of somatic cell mutations. The mutations in the genome lead to changes in the proteome, which eventually control the process of cellular transformation. As a consequence of genetic and epigenetic alterations, a cancer antigenome^27^ is generated, leading to the expansion of tumour antigen-specific T cells. The spectrum of the cancer antigenome includes non-mutated “self-antigens” that are the consequences of the tissue-specific or transformation-induced expression profiles of tumour cells and “neo-antigens” that arise as a direct consequence of somatic mutations within tumour cells.

In the present study, the diversity and frequency distribution of T-cell clones did not change during gastric tumourigenesis (Supplementary Figs S1 and S2; Fig. 1). However, the overlap of T-cell clones between gastric lesions and adjacent tissues decreased during malignant progression (Figs 2 and 3). Moreover, correlation analysis of the highly expanded common T-cell clones between gastric lesions and adjacent tissues revealed that the frequency similarity of the remaining common T-cell clones decreased sharply during the stages of tumourigenesis (Fig. 4). These results suggested that tissue-infiltrating lymphocytes in GPLs and EGC may undergo a selective antigen-driven clonal expansion.

Differences in infiltrating TCR repertoires between normal and cancer tissues have been reported in several studies^22–26^. However, none of these studies have linked this phenomenon to specific genes. In our study, using integrative analysis of gene expression profiles and TVI, we identified 788 genes closely related to changes in the TCR repertoire during gastric carcinogenesis. Finally, through network construction and module detection, we found an 11-gene module that was significantly correlated with the overall survival of GC patients (Figs 5 and 6). Among these genes, *CXCR5* and *CXCL13* have been reported to be closely related to Helicobacter pylori-induced inflammation^28, 29^. *CXCR1*, *CXCR2* and *CXCR4* are involved in the transition of chronic inflammation in the upper gastrointestinal tract to neoplasia^30^; additionally, *CXCR2* and *CXCR4* are independent prognostic predictors for GC patients^31–33^. Activation of *FPR2* induces the malignant behaviours of GC cells^34^. However, the remaining 5 genes have yet not been reported in GC. The functions of these genes need to be further investigated, and additional efforts are required to elucidate the mechanisms that lead to the changes in the TCR repertoire during gastric tumourigenesis. Our results provide a new strategy to discover potential prognostic biomarkers by integrating analysis of TCR repertoire changes in the progression of cancer and the local molecular phenotype.

In conclusion, for the first time, our study assessed the infiltrating TCR repertoires of GPLs (including LGIN and HGIN), EGC and matched adjacent tissues to elucidate the multistage heterogeneity of tissue-infiltrating TCR repertoire during gastric malignant transformation. Moreover, we determined the association between the degree of variation in the TCR repertoires and the expression profiles of corresponding gastric tissues and identified genes that may participate in the interaction between the mutated cells and immune system. Although the effects of these genes on the behaviours of TILs and cancer cells remain unclear and the results presented here require further exploration, our study reports a novel way of discovering biomarkers for GC prognosis, and improves our understanding of immune responses during carcinogenesis.

## Methods

### Sample collection and RNA isolation

A total of 41 gastric tissue samples from 19 patients, including 3 adjacent mucosa-LGIN-HGIN progression cascade samples and 16 paired samples (4 LGIN, 6 HGIN, 6 EGC and matched adjacent tissues), were obtained through upper magnifying chromoendoscopic targeted biopsy at the Department of Gastroenterology of the Peking Union Medical College Hospital (PUMCH) from 2011 to 2015. All tissue samples were incubated in RNA*later*® solution (Invitrogen, CA, USA) overnight at 4 °C and then stored at −80 °C. The pathological diagnosis was performed by two independent and experienced pathologists who were blinded to the study conditions. Samples that satisfied the diagnostic criteria for precancerous and neoplastic histology (abnormal cells > 80%) were enrolled. All adjacent tissue samples used in our study were diagnosed as chronic superficial gastritis, based on the Sydney classification. This study was reviewed and approved by the Ethics Committee of PUMCH, and all of the patients provided written informed consent. All the methods were carried out in accordance with the approved guidelines.

RNA was extracted from the whole biopsy specimen (6mm × 2.5mm × 3mm in size) using an RNeasy Mini Kit (Qiagen, MD, United States), and the concentration and RNA integrity were then determined using a NanoDrop ND-1000 spectrophotometer (NanoDrop Technologies, Wilmington, USA) and an Agilent 2100 Bioanalyzer (Agilent, CA, United States), respectively. RNA samples exhibiting an RNA integrity number (RIN) greater than 6.5 were included in the study.

### TCRβ sequencing and data analysis

The TCRβ library was obtained via three nested PCR reactions using previously described primers^35^ (Supplementary Table S4). The reaction protocol was modified from the ARM-PCR procedure^36, 37^. First, TCRβ-specific cDNA was reverse-transcribed from 500 ng of the total RNA using ProtoScript II Reverse Transcriptase (NEB, UK) and the primer TRBCRo. For the first PCR step, cDNA templates were amplified using a multiplex PCR 5x Master Mix (NEB, UK) with multiple V region primers of TCRβ chains (Vβ) (TRBV1Fo-TRBV30Fo) and a constant region primer of TCRβ chains (Cβ) (TRBCRo) (Supplementary Table S4) in a volume of 25 μl and the following thermocycling protocol: 95 °C for 3 min; 10 cycles of 95 °C for 30 sec, 60 °C for 2 min, and 68 °C for 1 min; and 68 °C for 5 min. The second PCR step was performed in a volume of 25 μl containing 2 μl of the first PCR product, the primers TRBV1Fi-TRBV30Fi of Vβ and TRBCRi of Cβ (Supplementary Table S4), NEB multiplex PCR master mix and H_2_O. The cycling conditions were identical to those of the first step. For the third PCR reaction prior to TCR sequencing, barcodes were incorporated to facilitate sequencing on the Illumina HiSeq. 2000 platform (paired-end, 250 bp). Briefly, 2 μl of the second PCR product was used as a template for the third 50-μl PCR reaction, and amplification was performed using Deep VentR (exo-) DNA polymerase (NEB, UK) for 25 cycles using the primers SuperF and SuperR. After a total of 45 PCR cycles, the final products were separated on a 1.2% agarose gel, and bands of approximately 250 to 500 bp were excised and gel purified using a QIAquick gel extraction kit (Qiagen, MD, United States). This purified product was then sequenced.

The raw data were cleaned using Trimmomatic v0.33^38^. We then used FLASH v1.2.11^39^ to merge the paired reads and to obtain the complete sequences of the TCRβ CDR3 regions. MiTCR^40^ was used to assign the rearranged mRNA sequences to their germline V, D, and J counterparts. The basic statistical and diversity analyses (calculation of ShannonDI)^41^ of the T-cell clones were accomplished using a post-alignment analysis tool, VDJtools v1.0.0^42^. All raw data of TCR sequencing are available from the SRA database (accession number SRP091344).

### Gene expression microarray

The RNA samples were analysed using the Agilent SurePrint G3 Human GE v2 8 × 60 K Microarray (G4851B). All samples were labelled, hybridized, and washed according to the manufacturer’s instructions. The slides were scanned using the Agilent SureScan Microarray Scanner (G2600D) and were extracted with Agilent Feature Extraction Software v10.5.1.1. The raw data were preprocessed and normalized with GeneSpring GX v12.6.1 (Silicon Genetics, CA, USA) using its default settings. The raw data and normalized data from 22 GPLs and EGC tissues were submitted to the GEO database under accession number GSE87666.

### Identifying the prognostic value of TVI related genes

The TVI was used to represent the degree of variation in the TCR repertoire during gastric tumourigenesis. The TVI is defined as:

1 $$TVI=2-F\times (1+C)$$

where *F* is the cumulative frequency of common T-cell clones between TOP100 of gastric lesions and the TCR repertoires of the corresponding adjacent tissues, and *C* is the Spearman’s rank correlation coefficient of “the top 100 common T-cell clones” between gastric lesions and the corresponding adjacent tissues. TVI values range from 0 to 2.

The STRING v10 database was used to screen PPI pairs, and the cutoff criteria were text mining > 200 and combined score > 400 (http://string.embl.de/). Spearman’s rank correlation test was applied to filter PPI pairs and identify TVI-related genes. The network was obtained and visualized using Cytoscape 3.2.1. Network cluster detection was performed based on CFinder 2.0.6, and DAVID was applied to analyse the GO enrichment of gene functions (https://david.ncifcrf.gov/).

### Statistical analysis

A paired-sample t-test was used to assess differences in the diversity of T-cell clones between matched adjacent tissues and LGIN, HGIN or EGC tissues. Differences among patients in the LGIN, HGIN and EGC groups were analysed using one-way ANOVA and the post hoc Tukey multiple comparison test. P-values < 0.05 were considered to indicate a significant difference. All analyses were performed using SPSS v17.0 (SPSS, Chicago, IL, USA). The data are presented as the mean ± SEM. Common T-clones refers to common T-cell clones detected between GPLs/EGC and matched adjacent tissues. The T-cell clone overlap ratio between GPLs/EGC and matched adjacent tissues was defined as the number of shared unique TCRβ reads divided by the number of unique reads detected in GPLs/EGC. The cumulative frequencies of common T-cell clones were calculated as frequencies from GPLs/EGC. Spearman’s rank correlation test was conducted using the R package “stat”, and a correlation was considered statistically significant if the false-discovery rate (FDR) adjusted p value was less than 0.05. Unsupervised hierarchical clustering analysis was performed using the R package “ape”. Survival curves were obtained using the Kaplan-Meier method and examined with the log-rank test using the R package “survival”. The RNA-seq data and corresponding clinical information, which were available for 388 GC samples, were downloaded from TCGA database using R Package “TCGA2STAT”.

## Electronic supplementary material

A novel signature for stratifying the molecular heterogeneity of the tissue-infiltrating T-cell receptor repertoire reflects gastric cancer prognosis
