# Supplementary material for: A novel signature for stratifying the molecular heterogeneity of the tissue-infiltrating T-cell receptor repertoire reflects gastric cancer prognosis
Source: Sci Rep. 2017 Aug 10;7:7762. doi: 10.1038/s41598-017-08289-z (PMC5552765; doi:10.1038/s41598-017-08289-z)
Supplement: Supplementary file 1 — A novel signature for stratifying the molecular heterogeneity of the tissue-infiltrating T-cell receptor repertoire reflects gastric cancer prognosis [file 41598_2017_8289_MOESM1_ESM.pdf]

Title: A novel signature for stratifying the molecular heterogeneity of the tissue-infiltrating T-cell receptor repertoire reflects gastric cancer prognosis.

Authors: Manchao Kuang[1*], Jieyao Cheng[2*], Chengli Zhang[1*], Lin Feng[1*], Xue Xu[2], Yajing Zhang[1], Ming Zu[2], Jianfang Cui[2], Hang Yu[2], Kaitai Zhang[1], Aiming Yang[2] & Shujun Cheng[1]

Supplementary Figure S1. The cumulative frequencies of the TOP100 in each sample. The frequencies did not significantly differ between adjacent tissues and LGIN (A), HGIN (B) or EGC (C). Two-tailed paired t-test, $p > 0.05$.

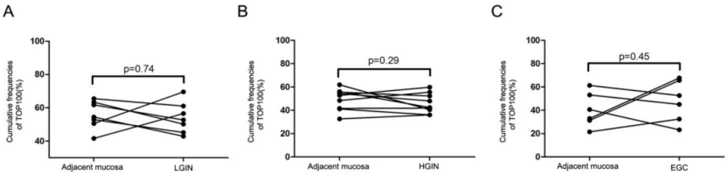

Supplementary Figure S2. The diversity of TCRβ repertoires in each sample represented by log2(ShannonDI) values. It showed no significant difference between adjacent tissues and LGIN (A), HGIN (B) or EGC (C). Two-tailed paired t-test, $p > 0.05$.

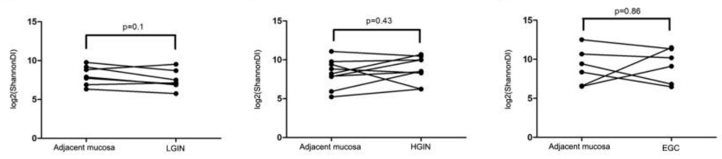

Supplementary Figure S3. Scattergram of node degrees in the co-expression network. The co-expression network was scale-free.

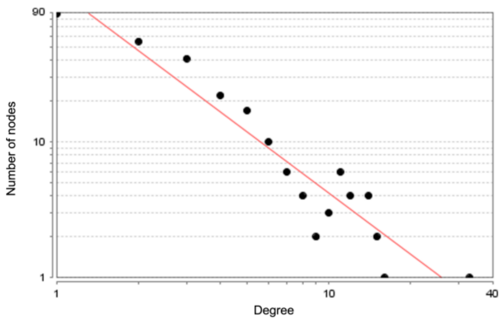

Supplementary Table S1. Statistical TCRβ sequencing data for each patient of different pathological stages.

Sample ID	sample type	Total TCRβ reads	Unique TCRβ reads	ShannonDI
Patient 1	Adjacent tissue	2284221	41931	875.48
	LGIN	2232415	33280	419.46
	HGIN	2722901	42137	1014.78
Patient 2	Adjacent tissue	2428738	45766	454.89
	LGIN	2764097	54837	737.17
	HGIN	2723911	50449	317.16
Patient 3	Adjacent tissue	2804572	48458	238.34
	LGIN	2885123	41722	117.50
	HGIN	3380530	52772	353.82
Patient 4	Adjacent tissue	3274798	35335	80.54
	LGIN	2670538	32048	53.93
Patient 5	Adjacent tissue	3610672	43914	119.21
	LGIN	2843396	40796	142.02
Patient 6	Adjacent tissue	3636481	56050	582.33
	LGIN	2821554	36435	182.11
Patient 7	Adjacent tissue	2956367	38008	211.72
	LGIN	3435932	42556	127.49
Patient 8	Adjacent tissue	2489492	36979	229.73
	HGIN	2776204	54889	1025.07
Patient 9	Adjacent tissue	3761614	58791	676.34
	HGIN	3702171	35644	75.20
Patient 10	Adjacent tissue	2920991	49438	295.72
	HGIN	2615922	61653	1649.64

Patient 11	Adjacent tissue	3637946	78550	2175.98
	HGIN	3265505	60143	1419.87
Patient 12	Adjacent tissue	3610903	33259	37.52
	HGIN	3195160	31812	75.77
Patient 13	Adjacent tissue	3176221	33665	60.79
	HGIN	4903297	62002	368.66
Patient 14	Adjacent tissue	3735499	40065	96.42
	EGC	3074936	72491	2884.51
Patient 15	Adjacent tissue	3092901	77901	681.57
	EGC	3285969	42242	112.96
Patient 16	Adjacent tissue	2619517	56283	1624.10
	EGC	3778274	47369	1163.81
Patient 17	Adjacent tissue	2814506	43504	328.44
	EGC	3540150	38742	88.62
Patient 18	Adjacent tissue	3999327	41138	92.20
	EGC	4161827	57771	548.92
Patient 19	Adjacent tissue	4408270	101029	5841.36
	EGC	4148965	78358	2574.10

Supplementary Table S2. The TCRβ clones were categorized based on differences in frequency in each patient of different pathological stages.

**Sample ID**	**sample type**	**TCRβ (%)**					
		**<0.0001%**	**0.0001-0.001%**	**0.001-0.01%**	**0.01-0.1%**	**0.1-1%**	**>1%**
Patient 1	Adjacent tissue	78.04	11.10	8.30	2.23	0.30	0.029
	LGIN	81.68	10.64	4.83	2.41	0.40	0.039
	HGIN	79.94	11.14	5.75	2.76	0.39	0.017
Patient 2	Adjacent tissue	80.72	12.28	4.72	2.00	0.25	0.02
	LGIN	82.84	10.25	4.76	1.89	0.25	0.018
	HGIN	86.20	10.28	1.78	1.42	0.28	0.034
Patient 3	Adjacent tissue	74.57	16.93	6.83	1.48	0.15	0.023
	LGIN	78.23	16.55	3.40	1.58	0.20	0.026
	HGIN	86.60	9.21	2.20	1.71	0.24	0.027
Patient 4	Adjacent tissue	82.64	13.17	2.31	1.66	0.17	0.042
	LGIN	78.62	17.69	2.01	1.42	0.23	0.034
Patient 5	Adjacent tissue	84.04	12.77	1.84	1.11	0.20	0.032
	LGIN	78.35	16.48	3.76	1.16	0.21	0.037
Patient 6	Adjacent tissue	86.72	7.63	3.62	1.80	0.20	0.03
	LGIN	82.08	13.29	2.91	1.44	0.24	0.049
Patient 7	Adjacent tissue	81.63	13.31	3.20	1.54	0.28	0.042

	LGIN	85.68	10.16	2.66	1.25	0.23	0.031
Patient 8	Adjacent tissue	79.09	13.93	5.00	1.71	0.24	0.041
	HGIN	73.12	14.22	10.49	1.99	0.17	0.022
Patient 9	Adjacent tissue	87.22	7.53	3.27	1.72	0.24	0.019
	HGIN	77.73	16.19	4.35	1.52	0.19	0.017
Patient 10	Adjacent tissue	75.72	15.79	6.62	1.68	0.16	0.02
	HGIN	76.64	12.09	8.96	2.14	0.16	0.013
Patient 11	Adjacent tissue	85.23	7.45	5.10	2.05	0.17	0.0038
	HGIN	86.32	6.85	4.29	2.27	0.27	0.012
Patient 12	Adjacent tissue	76.36	19.03	3.58	0.91	0.10	0.03
	HGIN	80.46	13.30	3.93	2.07	0.22	0.028
Patient 13	Adjacent tissue	79.35	15.52	3.69	1.21	0.21	0.03
	HGIN	85.95	9.30	3.19	1.35	0.19	0.018
Patient 14	Adjacent tissue	83.12	10.69	4.40	1.60	0.17	0.02
	EGC	78.26	9.64	10.11	1.88	0.12	0.0028
Patient 15	Adjacent tissue	68.81	20.98	8.84	1.30	0.068	0.0064
	EGC	81.75	12.05	4.93	1.10	0.13	0.04
Patient 16	Adjacent tissue	74.70	12.89	9.63	2.63	0.14	0.016
	EGC	75.61	12.56	8.63	2.93	0.26	0.0084
Patient 17	Adjacent tissue	78.87	15.90	3.81	1.18	0.20	0.034
	EGC	84.51	11.77	2.06	1.47	0.17	0.027
Patient 18	Adjacent tissue	81.09	12.87	4.72	1.11	0.18	0.029
	EGC	83.58	8.68	5.93	1.63	0.16	0.019
Patient 19	Adjacent tissue	75.60	11.10	11.54	1.69	0.073	0.003
	EGC	77.37	10.31	10.39	1.80	0.13	0.0077

Supplementary Table S3. GO enrichment analysis of the 11-gene module.

GO accession	GO term	Genes	FDR
GO:0006954	inflammatory response	7	2.77E-05
GO:0006935	chemotaxis	6	5.11E-06
GO:0007204	positive regulation of cytosolic calcium ion concentration	6	8.21E-06
GO:0070098	chemokine-mediated signaling pathway	5	6.77E-05

Supplementary Table S4. TCR sequencing primers.

Primer name	Sequence
TRBCRo	GTGTGGCCTTTTGGGTGTGG
TRBV1Fo	AATGAAACGTGAGCATCTGG
TRBV2Fo	GTGTCCCCATCTCTAATCAC
TRBV3Fo	TATGTATTGGTATAAACAGG
TRBV4Fo	GTCTTTGAAATGTGAACAAC
TRBV5Fo	GATCAAAACGAGAGGACAGC
TRBV6aFo	GTGTGCCCAGGATATGAACC
TRBV6bFo	CAGGATATGAGACATAATGC
TRBV7Fo	CTCAGGTGTGATCCAATTTC
TRBV9Fo	GAGACCTCTCTGTGTACTGG
TRBV10Fo	GGAATCACCCAGAGCCCAAG
TRBV11Fo	CCTAAGGATCGATTTTCTGC
TRBV12Fo	AGGTGACAGAGATGGGACAA
TRBV13Fo	CTATCCTATCCCTAGACACG
TRBV14Fo	AGATGTGACCCAATTTCTGG
TRBV15Fo	TCAGACTTTGAACCATAACG
TRBV16Fo	TATTGTGCCCCAATAAAAGG
TRBV17Fo	ATCCATCTTCTGGTCACATG
TRBV18Fo	GCAGCCCAATGAAAGGACAC
TRBV19Fo	TGAACAGAATTTGAACCACG
TRBV20Fo	TCGAGTGCCGTTCCCTGGAC
TRBV21Fo	GCAAAGATGGATTGTGTTCC
TRBV23Fo	CATTTGGTCAAAGGAAAAGG
TRBV24Fo	ATGCTGGAATGTTCTCAGAC
TRBV25Fo	CTCTGGAATGTTCTCAAACC
TRBV26Fo	CCCAGAATATGAATCATGTT
TRBV27Fo	TTGTTCTCAGAATATGMCC
TRBV28Fo	ATGTGTCCAGGATATGGACC
TRBV29Fo	TCACCATGATGTTCTGGTAC
TRBV30Fo	TGTGGAGGGAACATCAAACC
TRBCRi	TGGAGTTCAGACGTGTGCTCTTCCGATCTTCTGATGGCTCAAACACAGC
TRBV1Fi	**TCTTTCCCTACACGACGCTCTTCCGATCT**CATTGAAAACAAGACTGTGC
TRBV2Fi	**TCTTTCCCTACACGACGCTCTTCCGATCT**TGAAATCTCAGAGAAGTCTG
TRBV3Fi	**TCTTTCCCTACACGACGCTCTTCCGATCT**CTCTAAGAAATTTCTGAAGA
TRBV4Fi	**TCTTTCCCTACACGACGCTCTTCCGATCT**GGAGCTCATGTTTGTCTACA
TRBV5aFi	**TCTTTCCCTACACGACGCTCTTCCGATCT**CAGGGGCCCCAGTTTATCTT

TRBV5bFi	**TCTTTCCCTACACGACGCTCTTCCGATCT**GAAACARAGGAAACTTCCCT
TRBV6aFi	**TCTTTCCCTACACGACGCTCTTCCGATCT**GGTATCGACAAGACCCAGGC
TRBV6bFi	**TCTTTCCCTACACGACGCTCTTCCGATCT**TAGACAAGATCTAGGACTGG
TRBV7aFi	**TCTTTCCCTACACGACGCTCTTCCGATCT**TCTAATTTACTTCCAAGGCA
TRBV7bFi	**TCTTTCCCTACACGACGCTCTTCCGATCT**TCCCAGAGTGATGCTCAACG
TRBV7cFi	**TCTTTCCCTACACGACGCTCTTCCGATCT**ACTTACTTCAATTATGAAGC
TRBV7dFi	**TCTTTCCCTACACGACGCTCTTCCGATCT**CCAGAATGAAGCTCAACTAG
TRBV9Fi	**TCTTTCCCTACACGACGCTCTTCCGATCT**CTCATTCAGTATTATAATGG
TRBV10Fi	**TCTTTCCCTACACGACGCTCTTCCGATCT**GACATGGGCTGAGGCTGATC
TRBV11Fi	**TCTTTCCCTACACGACGCTCTTCCGATCT**ACTCTCAAGATCCAGCCTGC
TRBV12aFi	**TCTTTCCCTACACGACGCTCTTCCGATCT**TGCAGGGACTGGAATTGCTG
TRBV12bFi	**TCTTTCCCTACACGACGCTCTTCCGATCT**GTACAGACAGACCATGATGC
TRBV13Fi	**TCTTTCCCTACACGACGCTCTTCCGATCT**AAGATGCAGAGCGATAAAGG
TRBV14Fi	**TCTTTCCCTACACGACGCTCTTCCGATCT**AGTCTAAACAGGATGAGTCC
TRBV15Fi	**TCTTTCCCTACACGACGCTCTTCCGATCT**AAAGATTTTAACAATGAAGC
TRBV16Fi	**TCTTTCCCTACACGACGCTCTTCCGATCT**AATGTCTTTGATGAAACAGG
TRBV17Fi	**TCTTTCCCTACACGACGCTCTTCCGATCT**AACATTGCAGTTGATTCAGG
TRBV18Fi	**TCTTTCCCTACACGACGCTCTTCCGATCT**AATATCATAGATGAGTCAGG
TRBV19Fi	**TCTTTCCCTACACGACGCTCTTCCGATCT**TTTCAGAAAGGAGATATAGC
TRBV20Fi	**TCTTTCCCTACACGACGCTCTTCCGATCT**GATGGCAACTTCCAATGAGG
TRBV21Fi	**TCTTTCCCTACACGACGCTCTTCCGATCT**CGCTGGAAGAAGAGCTCAAG
TRBV23Fi	**TCTTTCCCTACACGACGCTCTTCCGATCT**GAATGAACAAGTTCTTCAAG
TRBV24Fi	**TCTTTCCCTACACGACGCTCTTCCGATCT**GTCAAAGATATAAACAAAGG
TRBV25Fi	**TCTTTCCCTACACGACGCTCTTCCGATCT**TAATTCCACAGAGAAGGGAG
TRBV26Fi	**TCTTTCCCTACACGACGCTCTTCCGATCT**ATTCACCTGGCACTGGGAGC
TRBV27Fi	**TCTTTCCCTACACGACGCTCTTCCGATCT**TGAGGTGACTGATAAGGGAG
TRBV28Fi	**TCTTTCCCTACACGACGCTCTTCCGATCT**AAAAGGAGATATTCCTGAGG
TRBV29Fi	**TCTTTCCCTACACGACGCTCTTCCGATCT**CTGGACAGAGCCTGACACTG
TRBV30Fi	**TCTTTCCCTACACGACGCTCTTCCGATCT**TTCTACTCCGTTGGTATTGG
SuperF	AATGATACGGCGACCACCGAGATCTACACTCTTTCCCTACACGACG
SuperR	CAAGCAGAAGACGGCATACGAGAT-barcode-GTGACTGGAGTTCAGACGTGTG

*Common sequences are indicated in bold.
